# Oncolytic Virus Immunotherapy

**DOI:** 10.3390/cancers13153672

**Published:** 2021-07-22

**Authors:** Antonio Marchini, Carolina S. Ilkow, Alan Melcher

**Affiliations:** 1 Laboratory of Oncolytic Virus Immuno-Therapeutics, Luxembourg Institute of Health, 84 Val Fleuri, L-1526 Luxembourg, Luxembourg; 2 German Cancer Research Centre, Laboratory of Oncolytic Virus Immuno-Therapeutics, Im Neuenheimer Feld 242, 69120 Heidelberg, Germany; 3 Cancer Therapeutics Program, Ottawa Hospital Research Institute, Ottawa, ON K1H 8L6, Canada; cilkow@ohri.ca; 4 Department of Biochemistry, Microbiology and Immunology, University of Ottawa, Ottawa, ON K1H 8M5, Canada; 5 Institute of Cancer Research, London SW3 6JB, UK; alan.melcher@icr.ac.uk

Oncolytic viruses (OVs) were originally developed as direct cytotoxic agents but have been increasingly recognised as a form of immunotherapy. Oncolytic viruses have now reached the stage of significant widespread clinical testing, with more than 40 OVs currently being evaluated for the treatment of various tumour entities [1]. The majority of past and ongoing clinical trials have been phase I studies evaluating the safety of the treatment as the primary (and most of the time only) end-point. Very few OVs entered more advance stages in the clinical development pipeline, and only one agent has been FDA- and EMA-approved, talimogene laherperepvec (T-VEC, an HSV encoding GMCSF), for intratumoural administration in advanced melanoma [2]. Whilst there is a wealth of encouraging early trial data confirming the safety of OVs across a number of viruses, tumour types and administration routes [1], more recent data from emerging larger, randomised studies have not been so encouraging. The last (and only) positive randomised phase 3 trial of an OV, testing T-VEC against subcutaneous granulocyte–macrophage colony-stimulating factor in melanoma, was published back in 2015 [2], and that study predated immunotherapy, which is now standard of clinical care in this disease. The next logical steps with T-VEC, combining the virus with checkpoint blockade, were initially encouraging with ipilimumab (an anti-CTLA4 antibody) [3], but the recent discontinuation of the randomised phase 3 of pembrolizumab (an anti-PD1) +/− T-Vec due to futility (Thousand Oaks, Calif., accessed on 2 February 2021 https://investors.amgen.com/news-releases/news-release-details/amgen-reports-fourth-quarter-and-full-year-2020-financial) has raised significant concerns about the long-term potential for the OV field in the clinic.

There have been other disappointing large, randomised trials. Vocimagene amiretrorepvec (TOCA 511) is a replicating retrovirus encoding a transgene for cytosine deaminase, which converts the prodrug 5-fluorocytosine into 5-fluorouracil. This failed in a study of over 400 patients, where viral injection into the resection cavity on first or second resection for high-grade glioma was randomised against standard of care treatment [4]. Then, pexastimogene devacirepvec (Pexa-Vec), a vaccinia virus again encoding GM-CSF, was also unsuccessful when tested after [5] or first line in combination with sorafenib in hepatocellular carcinoma.

Whilst there is no hiding from the disappointments of these studies, rather than abandoning the field, now is the time to reconsider and regroup. There are many drugs that fail on progression from early to randomised studies, but OVs represent an immune strategy rather than a single therapeutic, and so should not be abandoned en masse. Their greatest promise lies in ‘heating up’ an immunologically ‘cold’ tumour to prime for checkpoint blockade, and there are good translational clinical data that suggest that this can happen in patients [6,7]. There are a number of reasons why the large studies to date have been unsuccessful, including wrong choice of tumour type (for Pexa-Vec, advanced liver cancer patients’ often poor performance status makes altering the course of the disease notoriously difficult), clinical stage targeting (single agent pembrolizumab is too effective a single agent in limited metastatic melanoma for the addition of T-VEC to make a significant difference), and common problems seen on transitioning from early to later phase testing (in the TOCA511 study, patients had fewer cycles of treatment than in earlier trials). The key to further progress now is to better understand the immunobiology of OVs in patients via in-depth translational studies and to use this knowledge to inform careful development and progression of clinical trials in the most appropriate patient context.

We are proud and pleased to present this Special Issue of Cancers, “Oncolytic Virus immunotherapy” which includes 16 reviews written by many of the top oncolytic virus experts. There is a common message that comes across reading this issue, and it is one of justified optimism. The efficacy of OVs can be further improved, and the “second-generation” OV-based therapies once in the clinic may become a game changer in the history of cancer therapies for, e.g., pancreatic carcinoma, glioblastoma or lung cancer that still await effective treatment options. As our knowledge of the tumour cell, its microenvironment and components improves, the ideal of a “one size fits all” OV-based therapy becomes less real or attainable. Different individuals or cancers will need different approaches. Genetic engineering and arming of OVs and development of optimal combinatorial treatments must be carefully evaluated, taking into consideration the intra/inter patient heterogeneity of cancer and the complex interactions that cancer cells have with other components of the tumour microenvironment (resident and infiltrating non-transformed cells, secreted factors and extracellular matrix proteins). This complexity needs to be understood for every tumour entity and the (epi)genetic characteristics that distinguish it.

Oncolytic viruses are a very diverse group of “living drugs”, comprising viruses with very different biology and unique features. Every OV platform has strengths and weaknesses. Developing them further will exploit their positive aspects and mitigate the negative ones, considering the type and stage of cancer patients, route, schedule of administration, and the insurgence of neutralising antiviral immune responses that can reduce efficacy. To this end, it is also crucial to identify predictive biomarkers of response that suggest the most opportune OV treatment for each patient.

This Special Issue shows that while a real champion among OVs has not yet emerged, there are many great advances in the field that could lead to an improvement in therapeutic outcomes in the near future. A new wave of OV platforms are being developed thanks to the advances in genetic engineering and our improved understanding of the tumour ecosystem, which is allowing for the rational combination of OVs with other anti-cancer therapies. The importance of developing combination strategies that synergise against the tumour without leading to unwanted off-tumour effects is a common theme across the reviews. Müller et al. describe the community efforts for reovirus [8]; Burman et al. for Newcastle disease virus [9]; Engeland and Ungerechts for measles virus [10]; Angelova et al. for parvovirus [11]; and Malin and Kühnel [12] and Cunliffe et al. [13] for the adenovirus platforms. In addition to being “lysing machines”, OVs are “vehicles” that can deliver and express transgenes in the tumour ecosystem. Examples are given for the HSV platform by Vannini et al. [14]; for the adenovirus platform by Cunliffe et al. [13]; and, more generally, for the treatment of solid tumours by Jin et al. [15]. It is clear now that one avenue for improving the success of virotherapy resides in maximising the ability of OVs to harness the immune system to act against cancer, for example, through combination with other immunotherapies—especially immunocheckpoint blockers and adoptive cell therapy—or through the insertion of immunomodulatory transgenes into the virus genome. Combinatorial therapies of OVs and other treatment modalities are an active area of development, and, herein, Evgin and Vile [16], Kuryk et al. [17], Holbrook et al. [18] and Spiesschaert et al. [19] review the recent advances in this exciting field of research. Recent advances in genetic profiling of tumours are changing the way that we treat cancer patients. The latter is also impacting the way that we foresee the use of OVs in the near future. Both Fisher et al. [20] and Enrilich and Bacharach [21] describe the importance of understanding the tumour and its microenvironment for selecting the right OV platform for each cancer patient. Similarly, Stavrakaki et al. [22] discuss the importance of finding biomarkers to “personalise OVs” based on the tumour-specific characteristics. Finally, Kock et al. provide us with a comprehensive summary of oncolytic HSV-1 in its journey through the clinical arena [23].

We hope that the readers enjoy this Special Issue and that the OV scientific community continues working together towards the development of virotherapeutics that could positively impact the life of people living with cancer.
